# Does oral health-related quality of life of patients after solid organ transplantation indicate a response shift? Results of a systematic review

**DOI:** 10.1186/s12903-020-01350-w

**Published:** 2020-12-09

**Authors:** Gerhard Schmalz, Jens Garbade, Otto Kollmar, Dirk Ziebolz

**Affiliations:** 1 Department of Cariology, Endodontology and Periodontology, University of Leipzig, Liebigstr. 12, 04103 Leipzig, Germany; 2 University Department of Cardiac Surgery, Heart Center Leipzig, Leipzig, Germany; 3 Universitäres Bauchzentrum Basel, Universitätsspital Basel, Basel, Switzerland

**Keywords:** Oral health, Oral health-related quality of life, Solid organ transplantation

## Abstract

**Background:**

The physical oral health and dental behaviour of patients after solid organ transplantation (SOT) has repeatedly been reported as insufficient. The objective of this systematic review was to detect whether the oral health-related quality of life (OHRQoL) of patients after SOT is reduced compared to that of healthy individuals.

**Methods:**

A systematic literature search was performed by two independent individuals based on the PubMed, Web of Science and Scopus databases by using the following search terms: “transplantation” AND “oral health-related quality of life”. The findings were checked to determine eligibility, whereby publication prior to 31 October 2020, examination of adult patients (age at least 18 years) with SOT, reporting of an OHRQoL outcome and full text in English language were the prerequisites for inclusion in the qualitative analysis. Quality appraisal of the included studies was performed using the Agency for Healthcare Research and Quality methodology checklist.

**Results:**

Seven of 25 studies that examined patients after kidney (3), heart (2), liver (1) and lung transplantation (1) were included. Four studies included healthy controls, and five studies included a cohort of patients before transplantation for comparison. Clinical oral health examinations were heterogeneous between groups. The majority of studies (5/7) applied the short form of the “Oral Health Impact Profile” (OHIP 14) to assess OHRQoL. The OHIP 14 values ranged between 1.7 and 8.9 across studies, indicating an unaffected or just slightly reduced OHRQoL. Only one study found better OHRQoL in patients after SOT compared to a group before SOT, and one study confirmed worse OHRQoL of SOT recipients compared to a healthy control. Only two studies revealed an association between OHRQoL and oral health parameters. Furthermore, two studies each found a relationship between OHRQoL and general health-related quality of life or disease-related parameters.

**Conclusions:**

Patients after SOT show an unaffected or only slightly reduced OHRQoL, which was mainly independent of the insufficient oral status. This might indicate a shift in the perception threshold for oral diseases and conditions caused by the general health burden related to the SOT.

## Background

For many different end-stage organ diseases or conditions, solid organ transplantation (SOT) is an established and promising therapeutic approach that improves the physical and mental health of patients [1]. Due to successful surgical and posttransplant care, patient survival and morbidity have improved in recent decades, making quality of life issues increasingly relevant [2]. The quality of life of patients with SOT, including kidney (KTx), liver (LTx), lung (LuTx) and heart (HTx), is complex, and different physical, psychological, (psycho-)social and environmental parameters are of relevance [2]. Organ transplantation is a life-changing experience for patients and their relatives, making cognitive and emotional integration of the received organ a mandatory condition [3]. In general, health-related quality of life (HRQoL) is improved after SOT; however, anxiety, depression and psychosocial impairment are frequently occurring problems, making psychosocial support interventions recommended [2, 4, 5].

In addition to general disease-related parameters, HRQoL can be influenced by oral conditions [6]. In particular, tooth loss has the potential to affect the HRQoL of patients [6]. The specific subaspect of HRQoL in this context is oral health-related quality of life (OHRQoL), a multidimensional model including different physical and psychosocial issues [7]. This OHRQoL assesses the individual perception of a patient’s oral conditions and their perceived influence on oral function, psychosocial impacts, pain and orofacial appearance [7, 8]. Regularly, oral diseases, such as periodontitis, tooth loss (especially occluding pairs of teeth) or temporomandibular disorders, affect the OHRQoL [9–11].

The literature regarding the OHRQoL of patients after SOT is rare, and no systematic evaluation of different groups after SOT is available. However, it has already been documented that clinical oral health conditions are often poor in SOT recipients [12–16]. Thereby, reduced oral health behaviour, i.e., the low use of interdental cleaning devices and a switch from control- to complaint-oriented dental behaviour, can be observed [12–16]. These findings indicate that patients after SOT might concede their oral health situation as a low priority. This might be critical because oral diseases can constitute a risk for systemic infectious complications in these patients caused by their lifelong immunosuppressive medication [1, 17, 18]. Therefore, it would be of interest to determine whether the OHRQoL of SOT recipients is generally reduced and influenced by oral and/or disease-related parameters to draw conclusions on their appropriate multidisciplinary dental care.

This systematic review aimed to reveal the OHRQoL of patients after SOT, including KTx, LTx, LuTx and HTx. In addition to the OHRQoL of the patients in general, a second focus was to examine differences compared to healthy controls and/or patients before SOT (preTx). Furthermore, associations with HRQoL, oral health and disease-related parameters were considered. Therefore, the main objective was whether patients after SOT would show a reduced OHRQoL compared to healthy individuals.

## Methods

The authors followed the criteria established in the Preferred Reporting Items for Systematic Reviews and Meta-Analyses (PRISMA) guidelines for this review [19].

### Focused question

The PICO (patients, intervention, comparison, outcome) question of the article was whether patients after SOT would show a reduced OHRQoL. Accordingly, patients were individuals after SOT, while an intervention was not defined. The comparison was either a healthy control, patients before SOT or national reference values, and the outcome was an OHRQoL measurement. It was hypothesized that the OHRQoL of SOT patients would be nearly unaffected and not primarily associated with oral health parameters.

### Eligibility criteria

The following inclusion criteria were formulated previously: publication until 31 October 2020, examination of adult patients (age at least 18 years) with SOT, reporting of an OHRQoL measurement outcome and full text in English language.

### Search strategy

Two independent individuals performed this systematic review in November 2020. The literature search was based on the PubMed, Web of Science and Scopus databases, whereby the following search terms were applied: “transplantation” AND “oral health-related quality of life”. Additionally, a manual search was performed based on the references of the findings and similar articles. The respective findings were screened for eligibility.

### Data extraction

Within qualitative analysis, the following information was extracted from the included investigations:

- transplanted organ, year of publication, number of participants, study type, age, gender, disease duration.
- recruitment of a healthy control group or patients before SOT for comparison of OHRQoL findings.
- oral examinations and respective findings, if applicable.
- OHRQoL assessment, including form of measurement and results (mean values).
- potential relationship between OHRQoL and general and/or disease-related parameters.
- findings for subscales of the OHRQoL measurements, if applicable.

If patients were also part of previously published investigations, only the most recent study was included in the analysis. The whole process of systematic search and study selection as well as qualitative analysis was executed by two independent reviewers.

### Quality assessment

For quality appraisal of the seven included studies, the 11-item checklist as recommended by the Agency for Healthcare Research and Quality (AHRQ) for cross-sectional studies was applied [20]. To determine a total score for the assessment, the answers “no” or “unclear” were rated as 0, and the answer “yes” was rated as 1 point for each question. A total score of 0–3 indicated low quality, a score of 4–7 indicated moderate quality, and a score of 8–11 indicated high quality of the respective study. The quality assessment was independently conducted by the first author (GS) and the senior author (DZ). Any disagreements were discussed and resolved with the two other authors.

## Results

### Search findings

**Fig. 1:**
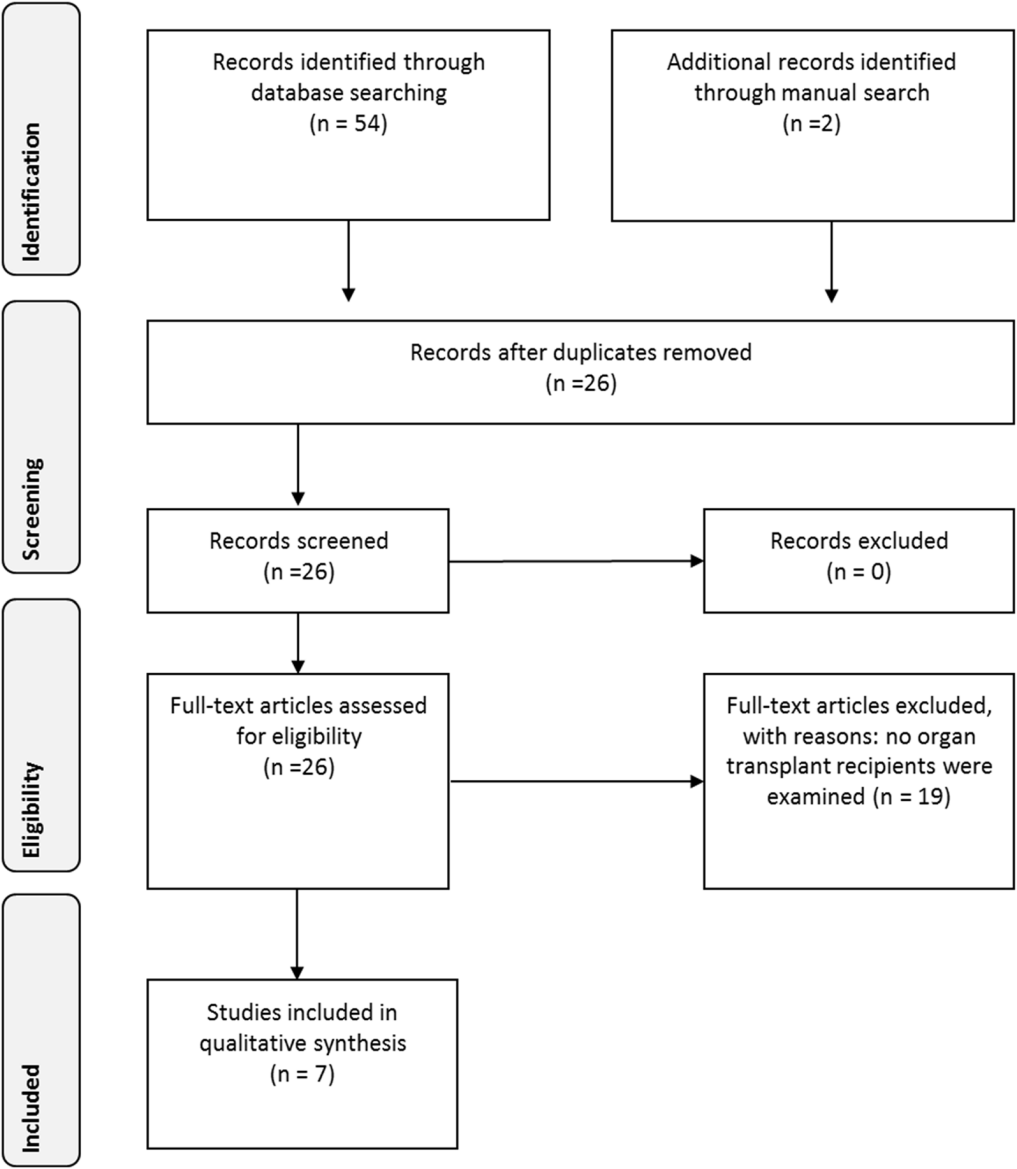
PRISMA diagram reflecting the study selection during the systematic review [42]

Based on the abovementioned search terms, 54 studies were found and were complemented by two more studies included by the manual search. After removal of duplicates and screening of the records, 26 full-text findings were checked for eligibility. While 19 studies did not meet the inclusion criteria (Additional file 1: Supplementary Table 1), seven clinical studies were included in the qualitative synthesis (Fig. 1).

**Table 1:** Quality assessment of the included studies following the Agency for Healthcare Research and Quality (ARHQ) methodology checklist [20]

Item	Segura-Saint-Gerons et al. [22]	Schmalz et al. [23]	Schmalz et al. [24]	Schmalz et al. [25]	Ruokonen et al. [21]	Schmalz et al. [26]	Oduncuoğlu et al. [27]
1) Define the source of information (survey, record review)	Yes	Yes	Yes	Yes	Yes	Yes	Yes
2) List inclusion and exclusion criteria for exposed and unexposed subjects (cases and controls) or refer to previous publications	Yes	Yes	Yes	Yes	Yes	Yes	Yes
3) Indicate time period used for identifying patients	No	No	Yes	No	Yes	No	Yes
4) Indicate whether or not subjects were consecutive if not population-based	Yes	Yes	Yes	Yes	Yes	Yes	Yes
5) Indicate if evaluators of subjective components of study were masked to other aspects of the status of the participants	No	No	No	No	No	No	No
6) Describe any assessments undertaken for quality assurance purposes (e.g., test/retest of primary outcome measurements)	Yes	Yes	Yes	Yes	Yes	Yes	Yes
7) Explain any patient exclusions from analysis	NA	NA	NA	NA	Yes	NA	NA
8) Describe how confounding was assessed and/or controlled	Yes	U	U	U	U	Yes	Yes
9) If applicable, explain how missing data were handled in the analysis	NA	NA	NA	NA	NA	NA	NA
10) Summarize patient response rates and completeness of data collection	Yes	Yes	Yes	Yes	Yes	Yes	Yes
11) Clarify what follow-up, if any, was expected and the percentage of patients for which incomplete data or follow-up was obtained	NA	NA	NA	NA	Yes	NA	NA
Total score	6	5	6	5	8	6	7

### Quality assessment

The findings of the quality appraisal are given in Table 1. The majority of studies, i.e., 6/7, indicated scores of moderate quality, while one study showed high quality [21].

### Characteristics of the included studies

Table 2 summarizes the characteristics of the included examinations. Three studies examined patients after KTx [21, 23, 27], two examined HTx [22, 26] and one study each examined patients after LuTx [24] or LTx [25]. All of the studies were performed in Europe, whereby most investigations took part in Germany. The number of included individuals ranged between 39 and 150 participants, with a mean age between 37.90 and 61 years. One study did not include a control group [22], one study included a healthy control group [24], two studies included a control group of patients before SOT [21, 26] and three studies included both a healthy control group and individuals before SOT for comparison [23, 25, 27].

**Table 2:** Overview of the study-specific parameters of the included studies. Values are presented as the mean values ± standard deviation, mean values (range) or percentages

Author, year	Treatment	Country	No. of patients	Study type	Subjects mean age in years	Time since Tx	Female (%)	Control group for OHRQoL	
								HC	preTx
Segura-Saint-Gerons et al. [22]	HTx	Spain	150	Monocentric cross-sectional	54.94 ± 14.56	30.7% < 5 years, 32% 5–10 years, 37.3% > 10 years	21.3%	No	No
Schmalz et al. [23]	KTx	Germany	39	Multicentric cross-sectional	56.51 ± 11.56	n/a	51.3%	Yes, n = 91, age: 58.31 ± 9.91 years, 65.9% female	Yes, HD n = 87, 37.9% Female, age: 60.98 ± 14.01
Schmalz et al. [24]	LuTx	Germany	60	Monocentric cross-sectional	54.03 ± 9.97	> 6 years: 58.3%	50%	Yes, n = 70, age: 55.44 ± 8.54 years, 63% female	No
Schmalz et al. [25]	LTx	Germany	47	Monocentric cross-sectional	46.6 ± 12.6	4.7 ± 3.9 years	42.6%	Yes, n = 75, 58.7% female, 57.1 ± 9.9	Yes, preLTx n = 24, 41.7% female, 54.4 ± 9.5 years
Ruokonen et al. [21]	KTx	Finland	51	Prospective observational	61 (31–86)	7.1 (1–11) years	33%	No	Yes, predialysis n = 144, 32.6% female, age 23–83 years
Schmalz et al. [26]	HTx	Germany	104	Monocentric cross-sectional	55.26 ± 12.16	6.8 ± 5.16 years	25%	No	Yes, HI n = 82, 54.90 ± 11.14 years, 15.9% female
Oduncuoğlu et al. [27]	KTx	Turkey	64	Monocentric cross-sectional	37.90 ± 10.30	1 year 18.8%, < 1–5 years 45.3%, > 5 years 35.9%	31.2%	Yes, n = 61, 37.10 ± 13.41, 65.6% female	Yes, HD n = 63, 40.98 ± 9.99 years, 38.1% female

*OHRQoL* oral health-related quality of life, *n/a*: not applicable, *HD* haemodialysis, *HI* heart insufficiency

### Oral health records and findings

Oral health examinations were heterogeneous between groups (Table 3). Six studies reported missing or remaining teeth of the patients, whereby the number of missing teeth varied between 3.69 and 9.5 [21, 23–27]. Of note, three studies defined 6 remaining teeth [23–25] and one study defined 10 remaining teeth as inclusion criteria [27]. The prevalence of moderate to severe periodontitis or periodontal treatment need was approximately high. Oral hygiene findings were only reported once [27].

**Table 3:** Examined oral health parameters and the main results of oral conditions if they were presented as the mean values ± standard deviation, means (range) or percentages in the included studies

Author, year	Tooth loss, remaining teeth, dentures	Dental diseases, caries, dental treatment need	Oral hygiene indices	Periodontal parameters, periodontal treatment need	Further oral health parameters
Segura-Saint-Gerons et al. [22]	38% denture wearing	n/a	n/a	n/a	n/a
Schmalz et al. [23]	M-T: 7.15 ± 6.21*	DMF-T: 17.41 ± 5.51, D-T 0.74 ± 0.43, F-T: 9.51 ± 4.23	n/a	87.2% Moderate to severe periodontitis	n/a
Schmalz et al. [24]	M-T: 8.17 ± 5.82*	DMF-T: 20.53 ± 5.09, D-T: 0.82 ± 1.85, F-T 11.55 ± 4.57	n/a	98% Moderate to severe periodontitis	n/a
Schmalz et al. [25]	M-T: 9.5 ± 5.6*	DMF-T: 21.6 ± 5.2, D-T: 1.5 ± 2.2, F-T 11.55 ± 4.57	n/a	74.5% moderate to severe periodontitis	n/a
Ruokonen et al. [21]	Remaining teeth: 21.7 ± 6.8	n/a	n/a	n/a	Xerostomia 40%, UWSF 0.32, SWSF: 0.95
Schmalz et al. [26]	M-T: 6.90 ± 7.27	DMF-T: 16.08 ± 7.11, dental treatment need: 16.3%	n/a	Periodontal treatment need: 85.6%	n/a
Oduncuoğlu et al. [27]	M-T: 3.69 ± 5.39**	DMF-T: 5.2 ± 5.8, D-T: 1.0 ± 1.62, F-T: 0.63 ± 1.52	GI: 1.33 ± 0.33, PI: 1.68 ± 0.4	PPD: 2.48 ± 0.6	n/a

*M-T* missing teeth, *D-T* decayed teeth, *F-T* filled teeth, *DMF-T* decayed-, missing- and filled teeth index, *PI* plaque index, *GI* gingival index, *PPD* periodontal probing depth, *UWS* unstimulated whole saliva, *SWS* stimulated whole saliva, *n/a* not applicable

*inclusion criterion: at least 6 remaining teeth

** inclusion criterion: at least 10 remaining teeth

### OHRQoL measurements and results

The majority of studies (5/7) reported the short form of the “Oral Health Impact Profile” (OHIP 14) [23–27]. One study reported the long form (OHIP 49) [22], one study presented a self-composed “oral health quality score” (OHQS) [21] and one investigation additionally included the OHRQoL-UK questionnaire (Table 4) [27]. The OHIP 14 values ranged between 1.70 and 8.9 points. Only one study found better OHRQoL in patients after SOT compared to a preTx group [27]. Similarly, only one study confirmed worse OHRQoL in SOT recipients than in healthy controls [25]. Two studies each revealed an association and/or correlation between OHRQoL and general health-related quality of life (HRQoL) [21, 26], oral health parameters [21, 22] or disease-related parameters [22, 27]. Three studies reported OHRQoL subscales, whereby different subscales were applied (Table 5) [22, 26, 27].

**Table 4:** Applied assessments for OHRQoL and relevant results for the included studies

Author, year	Assessment of OHRQoL	OHRQoL different compared to control		Association/correlation between OHRQoL and general HRQoL	Association/correlation between OHRQoL and oral health	Association and/or correlation between OHRQoL and disease-related parameters
		Better than preTx	Worse than healthy control (HC)			
Segura-Saint-Gerons et al. [22]	OHIP 49: 24.43	n/a	n/a	n/a	Number of daily tooth-brushing, dental visits in previous year	Gender
Schmalz et al. [23]	OHIP 14: 2.54 ± 3.68	No (HD OHIP 14: 2.46 ± 4.68)	No (HC OHIP 14: 1.52 ± 2.71)	n/a	No	No
Schmalz et al. [24]	OHIP 14: 1.70 ± 2.70	n/a	No (HC OHIP 14: 1.54 ± 2.86)	n/a	No	No
Schmalz et al. [25]	OHIP 14: 4.1 [1;0–5]	No (preLTx OHIP 14: 4.2 [1.5; 0–4.0]	Yes (HC OHIP 14: 1.4 [0; 0–2.0]	n/a	No	No
Ruokonen et al. [21]	75.1% maximum OHQS score	71.5%*	n/a	Correlation 15D with OHQS	PPD, PIBI, TDI, number of teeth, UWS	n/a
Schmalz et al. [26]	OHIP 14: 6.58 ± 6.40	No (HF OHIP G14: 5.54 ± 5.47)	n/a	Correlation PCS and MCS of SF-36 with OHIP 14	No	No
Oduncuoğlu et al. [27]	OHIP 14: 8.9 ± 9.6; OHRQoL-UK: 44.8 ± 10.5	Yes (HD OHIP 14: 12.28 ± 8.90)	no (HC OHIP 14: 9.0 ± 9.3)	n/a	n/a	Time since TX

*n/a* not applicable, *OHIP* oral health impact profile, *PCS* physical compound summary, *MCS* mental compound summary, *SF-36* short form 36 questionnaire, *PPD* periodontal probing depth, *UWS* unstimulated whole saliva, *PIBI* periodontal inflammatory burden index, *TDI* total dental index, *OHQS* oral health quality score, *HD* haemodialysis, *LTx* liver transplantation, *HF* heart failure, *15D* 15D questionnaire

*No significance testing reported

**Table 5:** Subscales of OHRQoL in the included studies. Because different questionnaires were used and several different options of subscales/dimensions exist, the available results are presented if available. The results are given as the mean values ± standard deviation or otherwise as percentages

Author, year, disease	Functional limitation	Physical pain	Psycho-social discomfort	Physical disability		Psycho-logical disability	Social disability	Handicap
*OHIP 14*								
Oduncuoğlu et al. KTx [27]	1.0 ± 1.5	1.7 ± 2.0	1.3 ± 1.9	1.0 ± 1.8		1.3 ± 1.7	1.7 ± 1.8	1.0 ± 1.5
	Oral function				Psychosocial impact			
Schmalz et al. HTx [26]	1.30 ± 2.40				2.04 ± 3.86			
*OHIP 49*								
Segura-Saint-Gerons et al. HTx [22]	6.5 ± 5.71	6.82 ± 6.72	3.42 ± 4.28	3.31 ± 5.04		2.31 ± 4.01	0.83 ± 2.34	1.24 ± 2.54

*OHIP* oral health impact profile, *KTx* kidney transplantation, *HTx* heart transplantation

## Discussion

This systematic review revealed seven clinical studies that examined the OHRQoL of patients after SOT. Only one study each found the OHRQoL of SOT recipients to be different compared to patients before SOT and healthy controls. Accordingly, it is difficult to evaluate whether the OHRQoL of these patients is reduced or impaired by their oral and/or systemic conditions. The majority of studies applied the OHIP 14, a valid and well-established questionnaire-based measurement that evaluates 14 different functional and psychosocial impairments that patients perceived with respect to their oral cavity (Additional file 1: Supplementary Table 2) [7, 28, 29]. Due to cross-cultural differences between different OHIP translations [29] and differences in patient groups and health systems between different countries, the comparability of the included studies might be limited. While no international reference values are available, the German reference can be used to estimate whether there is a reduction in the OHRQoL of patients after SOT. For OHIP 14, a reference value between 0 and 4 out of 56 points, whereby higher values indicate worse OHRQoL, can be stated based on the dentition of patients [30]. Three of the included studies, which were performed in Germany, are within this reference [23–25]. Two other studies using OHIP 14 presented slightly higher scores [26, 27]. Another study applied the OHIP 49, which is the long version of OHIP 14, of which a score between 5 and 15 out of 196 points can be seen as a reference [31]. Accordingly, the reported OHIP 49 lies slightly higher than the reference [22]. Although a general statement regarding the OHRQoL of SOT recipients is limited due to different patient cohorts (different organs, countries, mean age, oral status), the OHRQoL of these patients appears not or at most slightly reduced.

Several issues need to be discussed in this context. In general, the physical oral health findings in relation to the patients’ perception of their oral conditions seem contradictory. In a Turkish study, which applied OHIP 14, the worst OHRQoL was found [27]. This study had the lowest mean age and lowest number of missing teeth out of all included studies (Table 1). However, age and tooth loss regularly affect OHRQoL [9, 32, 33]. Accordingly, the reduced OHRQoL of this cohort of Turkish patients is surprising and might indicate a perception of oral health situation, which is not in line with the clinical situation. Because this study did not examine associations between oral health and OHRQoL of the patients, this factor remains speculative. In contrast, four German studies found the OHRQoL to be independent of insufficient dental and periodontal status [23–26]. These four studies found a high periodontitis prevalence or treatment need (Table 1). In regular cases, this should lead to an impairment of OHRQoL [10, 34]. Accordingly, the findings also argue for a patient´s perception of the oral health situation, which is not in line with the clinical situation. However, the included studies did not report in detail the extent or severity of periodontal diseases (e.g., tooth loosening, pronounced recession, active inflammation). Only one study, performed in Finland, revealed associations between physical oral findings and OHRQoL [21]. Therefore, OHRQoL was assessed by a self-composed questionnaire (OHQS), including questions on last dental check-up, toothbrushing, smoking or dry mouth [21]. This questionnaire is not comparable to regular OHRQoL assessment with the OHIP, which assesses different functional and psychosocial impacts of the patient and not oral behaviour (Additional file 1: Table 2). The study by Segura-Saint-Gerons et al. found associations between OHIP 49 and oral behaviour (tooth brushing and dental visits) but not with physical oral health [22]. Accordingly, although this conclusion must be interpreted with caution, the OHRQoL of patients after SOT, which is not or only slightly reduced, appears to be mainly independent of physical oral health conditions.

Two studies found a relationship between general HRQoL and OHRQoL [21, 26]. In general, HRQoL and OHRQoL are closely related in generally healthy individuals, where OHRQoL can be seen as a subaspect of the whole HRQoL [35]. However, specific diseases might affect this relationship to an unclear extent [36]. Furthermore, HRQoL can also be directly associated with oral health conditions [6]. This effect has not yet been considered in examinations of SOT recipients. It is well described that the general HRQoL of SOT recipients is impaired and that different important emotional and psychosocial issues are of relevance [2, 4, 5]. The general burden related to SOT, medication, psychological issues (acceptance of transplant, perceived relationship to donor) and comorbidities might affect the perception of other areas of life, e.g., oral health and dental behaviour. Based on the missing association between OHRQoL and the physical oral situation on the one hand and the association between OHRQoL and HRQoL on the other hand, a shift in patients’ oral health perception can be hypothesized.

**Fig. 2:**
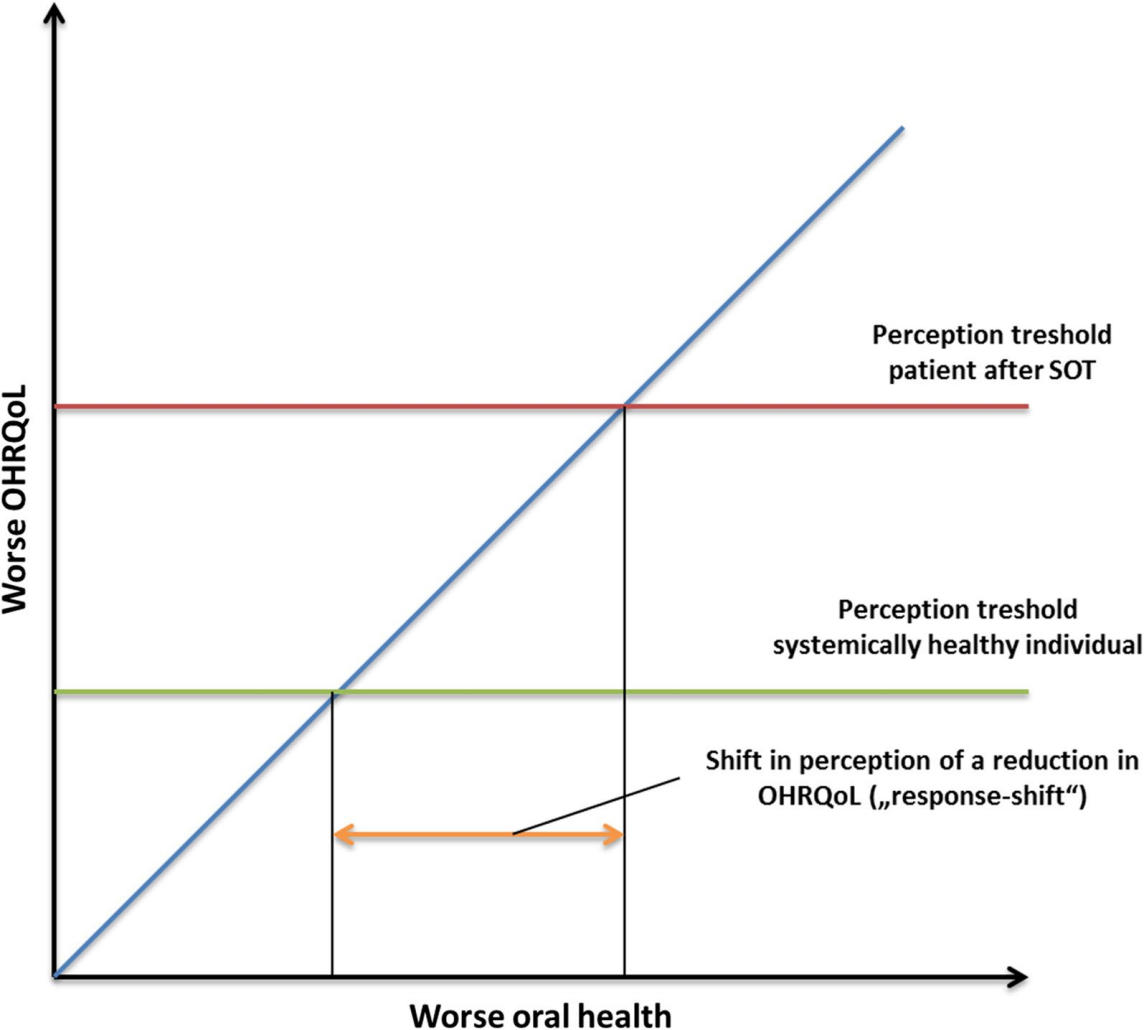
Change in the perception of the patients’ oral health situation due to the burden of SOT and related parameters, such as psychological, physical and social impairment

In this context, the “response shift theory”, as formulated by Sprangers and Schwartz in 1999, can be quoted [37]. This phenomenon describes a cognitive change in patients with severe chronic diseases, which leads to a postponement of the internal standard due to the accommodation of the status “chronic disease” [37]. Of course, the strict transferability of this model in the context of the OHRQoL of SOT recipients is limited; assessment and interpretation of the “response shift” phenomenon is difficult [38, 39]. Previously, “response shift” assessment was applied in the context of dentistry, especially with regard to prosthodontic rehabilitation, assessment of perceived treatment effects or dentine hypersensitivity [40–42]. However, these deliberations were only focused on oral disease or dental therapy measures. Based on the findings of this systematic review, it might be conceivable that the accommodation of the chronically diseased status of SOT recipients might affect their oral health perception. This might lead to a shift in the perception threshold for impairment in OHRQoL and possibly HRQoL caused by oral diseases and conditions. Therefore, patients might not perceive oral diseases, such as chronic periodontitis or several missing teeth, as impairments in their OHRQoL and might be affected only if acute dental issues, such as pain or extended tooth loss, appear (Fig. 2).

**Fig. 3:**
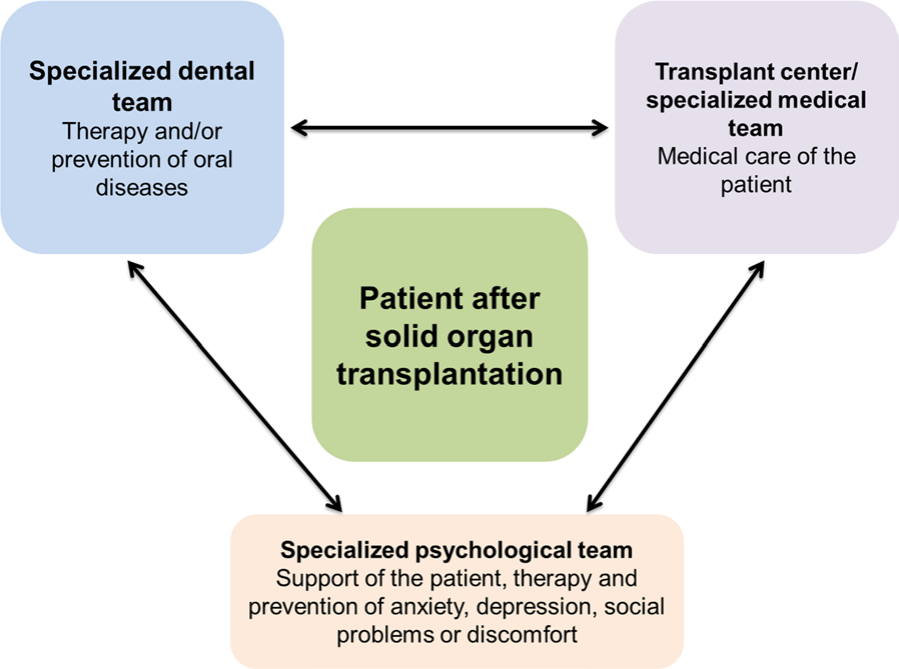
For sufficient, patient-oriented oral care of SOT recipients, a multidisciplinary team might be necessary to build awareness of the importance of healthy oral conditions

Of course, this is just a hypothesis based on the nearly unaffected OHRQoL of SOT recipients independently of their oral status. However, this might be of high practical relevance for dental care. If a patient does not feel impairment of his/her oral condition, this patient might not see the necessity to visit the dentist or to increase oral hygiene behaviour. This would explain the poor clinical oral health conditions and reduced oral health behaviour, i.e., the low use of interdental cleaning devices and a switch from control- to complaint-oriented dental behaviour of SOT recipients [12–16]. However, early dental rehabilitation and sufficient maintenance of SOT recipients are necessary to reduce the risk of systemic infections related to their lifelong immunosuppressive medication [1, 17, 18]. This early and prevention-oriented dental care seems to not work yet [43]. Therefore, multidisciplinary oral care appears necessary. Based on the current findings and formed hypothesis, the interdisciplinary team should include dental staff and transplant centres as well as psychological teams to build awareness of the importance of healthy oral conditions for these patients (Fig. 3).

This is the first systematic review on the OHRQoL of SOT recipients. It was executed according to the PRISMA statement [19] by two independently operating individuals. While based on the search findings, the clinically relevant hypothesis of a phenomenon, which is similar to a “response shift”, could be formed, several general methodological issues of the included studies should be recognized. The included studies had certain heterogeneity regarding country, transplanted organs, age, oral examinations and OHRQoL measurements. This is important because a direct comparison between the different organ groups is not possible. However, this is the first systematic insight into the perception of OHRQoL by recipients of different SOT, which revealed common findings of clinical relevance. Because few data are available, the focus on one single organ group currently does not make sense and justifies including the heterogeneous group of different SOT recipients. It is known that oral diseases regularly affect OHRQoL [9–11]. To assess the real influence of oral conditions on the OHRQoL of SOT recipients, profound oral examinations, including the extent and severity of physical oral health impairment, might be necessary. This might include the severity and activity of periodontitis considering the new classification [44] or the number of remaining functional occlusal pairs instead of only assessing the number of missing teeth [9]. Furthermore, standardized and validated instruments, e.g., OHIP 14, should be applied, and future studies should aim to reveal reference values for SOT recipients. Only one study reported on the validity of the OHIP for SOT recipients [27], which should be extended in future research in the field. The reporting and analysis of different subscales, such as oral function, psychosocial impact, pain or orofacial appearance, might increase the understanding of individual patient cases [8]. Moreover, HRQoL and disease-related parameters as well as psychological issues, such as anxiety and/or depression, need to be considered to allow a complex understanding of the OHRQoL of these patients. Furthermore, longitudinal studies are needed to prove the hypothesis of a “response shift”, for which valid methods should be used [38, 39]. In general, assessment of the OHRQoL of patients after SOT can help understand the complexity of this patient group and to develop dental special care, which could allow successful, patient-oriented and multidisciplinary therapy and disease prevention.

## Conclusions

Patients after SOT show an unaffected or slightly reduced OHRQoL, which is mainly independent of the insufficient oral status. A possible reason could be a shift in the perception threshold for oral diseases and conditions caused by the general health burden related to SOT. This might indicate the necessity for multidisciplinary dental care to (re)build patients’ awareness of the importance of oral health. Well-designed, longitudinal studies are needed to prove these conclusions.

## Supplementary information

**Additional file 1:** Supplementary Table 1 and Supplementary Table 2.

## Data Availability

All data generated or analysed during this study are included in this published article.

## Abbreviations

D-T: Number of decayed teeth
DMF-T: Decayed-, missing- and filled teeth index
F-T: Number of filled teeth
GI: Gingival index
HRQoL: Health-related quality of life
HTx: Heart transplantation
KTx: Kidney transplantation
LTx: Liver transplantation
LuTx: Lung transplantation
M-T: Number of missing teeth
OHIP: Oral health impact profile
OHRQoL: Oral health-related quality of life
PI: Plaque index
PICO: Patient, intervention, control, outcome
PPD: Periodontal probing depth
SOT: Solid organ transplantation
Tx: Transplantation
