# Supplementary material for: Does oral health-related quality of life of patients after solid organ transplantation indicate a response shift? Results of a systematic review
Source: BMC Oral Health. 2020 Dec 9;20:356. doi: 10.1186/s12903-020-01350-w (PMC7726902; doi:10.1186/s12903-020-01350-w)
Supplement: Supplementary file 1 — Additional file 1: Supplementary Table 1 and Supplementary Table 2. [file 12903_2020_1350_MOESM1_ESM.docx]

**Supplementary material**

**Supplementary Table 1**: List of excluded full-text articles with reason for exclusion

| **Author and year** | **Reason for exclusion** |
| --- | --- |
| *Gianetti et al. 2007 [S1]* | No patients with SOT were examined (dental avulsion) |
| *Wogelius et al. 2011 [S2]* | No patients with SOT were examined (survivors of childhood cancer) |
| *Kushner et al. 2008 [S3]* | No patients with SOT were examined (oral mucositis) |
| *Hassel et al. 2012 [S4]* | No patients with SOT were examined (oral squamous cell carcinoma) |
| *Landes et al. 2012 [S5]* | No patients with SOT were examined (patients with clefts after bone graft and implant treatment) |
| *Reissmann et al. 2013 [S6]* | No patients with SOT were examined (impact of bone graft harvesting for implant treatment on OHRQoL) |
| *Kuoppala et al. 2013 [S7]* | No patients with SOT were examined (patients with mandibular bone grafts) |
| *Tinoco-Araujo et al. 2015 [S8]* | No patients with SOT were examined (patients before haematopoietic stem cell transplantation) |
| *Rocha Dos Santos et al. 2017 [S9]* | No patients with SOT were examined (treatment of localized gingival recessions) |
| *Lins et al. 2017 [S10]* | No patients with SOT were examined (liver transplant candidates), no assessment of OHRQoL |
| *Tonetti et al. 2018 [S11]* | No patients with SOT were examined (treatment of adjacent gingival recession) |
| *Staudenmaier et al. 2018 [S12]* | No patients with SOT were examined (oral mucositis) |
| *Rodakowska et al. 2018 [S13]* | No patients with SOT were examined (chronic haemodialysis) |
| *Kumar et al. 2018 [S14]* | No patients with SOT were examined (patients with free fibula flap reconstructed mandibles) |
| *Wyrębek et al. 2018 [S15]* | No patients with SOT were examined (patients with free gingival graft) |
| *Garbade et al. 2020 [S16]* | No patients with SOT were examined (patients with left ventricular assist device) |
| *Schmalz et al. 2020 [S17]* | Systematic review on renal replacement therapy |
| *Vidigal et al. 2020 [S18]* | No patients after SOT were examined (paediatric liver transplant candidates) |
| *Stolze et al. 2020 [S19]* | Systematic review on patients with haematological malignancies |

SOT: solid organ transplantation, OHRQoL: oral health-related quality of life

**References Supplementary Table 1**

S1. Giannetti L, Murri A, Vecci F, Gatto R. Dental avulsion: therapeutic protocols and oral health-related quality of life. Eur J Paediatr Dent. 2007;8:69-75.

S2. Kushner JA, Lawrence HP, Shoval I, Kiss TL, Devins GM, Lee L, Tenenbaum HC. Development and validation of a Patient-Reported Oral Mucositis Symptom (PROMS) scale. J Can Dent Assoc. 2008;74:59.

S3. Wogelius P, Rosthøj S, Dahllöf G, Poulsen S. Oral health-related quality of life among survivors of childhood cancer. Int J Paediatr Dent. 2011;21:465-7.

S4. Hassel AJ, Danner D, Freier K, Hofele C, Becker-Bikowski K, Engel M. Oral health-related quality of life and depression/anxiety in long-term recurrence-free patients after treatment for advanced oral squamous cell cancer. J Craniomaxillofac Surg. 2012;40:e99-102.

S5. Landes CA, Bündgen L, Laudemann K, Ghanaati S, Sader R. Patient satisfaction after prosthetic rehabilitation of bone-grafted alveolar clefts with nonsubmerged ITI Straumann dental implants loaded at three months. Cleft Palate Craniofac J. 2012;49:601-8.

S6. Reissmann DR, Dietze B, Vogeler M, Schmelzeisen R, Heydecke G. Impact of donor site for bone graft harvesting for dental implants on health-related and oral health-related quality of life. Clin Oral Implants Res. 2013;24:698-705.

S7. Kuoppala R, Kainulainen VT, Korpi JT, Sándor GK, Oikarinen KS, Raustia A. Outcome of treatment of implant-retained overdenture in patients with extreme mandibular bone resorption treated with bone grafts using a modified tent pole technique. J Oral Maxillofac Surg. 2013;71:1843-51.

S8. Tinoco-Araujo JE, Orti-Raduan ES, Santos D, Colturato VA, Souza MP, Mauad MA, Saggioro TC, Bastos RS, da Silva Santos PS. Oral health-related quality of life before hematopoietic stem cell transplantation. Clin Oral Investig. 2015;19:2345-9.

S9. Rocha Dos Santos M, Sangiorgio JPM, Neves FLDS, França-Grohmann IL, Nociti FH Jr, Silverio Ruiz KG, Santamaria MP, Sallum EA. Xenogenous Collagen Matrix and/or Enamel Matrix Derivative for Treatment of Localized Gingival Recessions: A Randomized Clinical Trial. Part II: Patient-Reported Outcomes. J Periodontol. 2017;88:1319-28.

S10. Lins L, Aguiar I, Carvalho FM, Souza L, Sarmento V, Codes L, Bittencourt P, Paraná R, Bastos J. Oral Health and Quality of Life in Candidates for Liver Transplantation. Transplant Proc. 2017;49:836-40.

S11. Tonetti MS, Cortellini P, Pellegrini G, Nieri M, Bonaccini D, Allegri M, Bouchard P, Cairo F, Conforti G, Fourmousis I, Graziani F, Guerrero A, Halben J, Malet J, Rasperini G, Topoll H, Wachtel H, Wallkamm B, Zabalegui I, Zuhr O. Xenogenic collagen matrix or autologous connective tissue graft as adjunct to coronally advanced flaps for coverage of multiple adjacent gingival recession: Randomized trial assessing non-inferiority in root coverage and superiority in oral health-related quality of life. J Clin Periodontol. 2018;45:78-88.

S12. Staudenmaier T, Cenzer I, Crispin A, Ostermann H, Berger K. Burden of oral mucositis in stem cell transplant patients-the patients' perspective. Support Care Cancer. 2018;26:1577-84.

S13. Rodakowska E, Wilczyńska-Borawska M, Fryc J, Baginska J, Naumnik B. Oral health-related quality of life in patients undergoing chronic hemodialysis. Patient Prefer Adherence. 2018;12:955-61.

S14. Kumar VV, Srinivasan M. Masticatory efficiency of implant-supported removable partial dental prostheses in patients with free fibula flap reconstructed mandibles: A split-mouth, observational study. Clin Oral Implants Res. 2018;29:855-63.

S15. Wyrębek B, Górski B, Górska R. Patient morbidity at the palatal donor site depending on gingival graft dimension. Dent Med Probl. 2018;55:153-9.

S16. Garbade J, Rast J, Schmalz G, Eisner M, Wagner J, Kottmann T, Oberbach A, Lehmann S, Haak R, Borger MA, Binner C, Ziebolz D. Oral health and dental behaviour of patients with left ventricular assist device: a cross-sectional study. ESC Heart Fail. 2020;7:1273-81.

S17. Schmalz G, Patschan S, Patschan D, Ziebolz D. Oral health-related quality of life in adult patients with end-stage kidney diseases undergoing renal replacement therapy - a systematic review. BMC Nephrol. 2020;21:154.

S18. Vidigal EA, Abanto J, Haddad AE, Porta G, Alves FA, BÖnecker M. Oral health-related quality of life among pediatric liver transplant candidates. Braz Oral Res. 2020;34:e100.

S19. Stolze J, Vlaanderen KCE, Raber-Durlacher JE, Brand HS. The impact of hematological malignancies and their treatment on oral health-related quality of life as assessed by the OHIP-14: a systematic review. Odontology. 2020;108:511-20.

**Supplementary Table 2**: Questions of the short form of the oral health impact profile (OHIP 14) [28]. Each question can be answered on a scale between 0 (“never”) and 4 (“always”), whereby higher scores reflect worse OHRQoL.

| **Subscale** | **Question** |
| --- | --- |
| Functional limitation | Have you had trouble pronouncing any words because of problems with your teeth, mouth or dentures? |
| Functional limitation | Have you felt that your sense of taste has worsened because of problems with your teeth, mouth or dentures? |
| Physical pain | Have you had painful aching in your mouth? |
| Physical pain | Have you found it uncomfortable to eat any foods because of problems with your teeth, mouth or dentures? |
| Psychological discomfort | Have you been self-conscious because of your teeth, mouth or dentures? |
| Psychological discomfort | Have you felt tense because of problems with your teeth, mouth or dentures? |
| Physical disability | Has your diet been unsatisfactory because of problems with your teeth, mouth or dentures? |
| Physical disability | Have you had to interrupt meals because of problems with your teeth, mouth or dentures? |
| Psychological disability | Have you found it difficult to relax because of problems with your teeth, mouth or dentures? |
| Psychological discomfort | Have you been slightly embarrassed because of problems with your teeth, mouth or dentures? |
| Social disability | Have you been slightly irritable with other people because of problems with your teeth, mouth or dentures? |
| Social disability | Have you had difficulty doing usual jobs because of problems with your teeth, mouth or dentures? |
| Handicap | Have you felt that life in general was less satisfying because of problems with your teeth, mouth or dentures? |
| Physical disability | Have you been totally unable to function because of problems with your teeth, mouth or dentures? |
